# The alternative renin–angiotensin system in critically ill patients: pathophysiology and therapeutic implications

**DOI:** 10.1186/s13054-023-04739-5

**Published:** 2023-11-20

**Authors:** Bruno Garcia, Alexander Zarbock, Rinaldo Bellomo, Matthieu Legrand

**Affiliations:** 1 Department of Anesthesia and Peri-Operative Care, Division of Critical Care Medicine, University of California, San Francisco (UCSF), San Francisco, CA USA; 2 Department of Intensive Care, Centre Hospitalier Universitaire de Lille, Lille, France; 3 Experimental Laboratory of the Department of Intensive Care, Université Libre de Bruxelles, Brussels, Belgium; 4 Department of Anesthesiology, Intensive Care and Pain Medicine, University Hospital of Münster, Münster, Germany; 5 Department of Intensive Care, Austin Hospital, Melbourne, VIC 3084 Australia; 6 Australian and New Zealand Intensive Care Research Centre, Monash University, Melbourne, Australia; 7 Department of Critical Care, Melbourne Medical School, University of Melbourne, Melbourne, VIC Australia

**Keywords:** Shock, Acute lung injury, Acute respiratory distress syndrome, Acute kidney injury, Angiotensin-converting enzyme 2, Angiotensin-(1–7), Angiotensin II, Dipeptidyl-peptidase 3

## Abstract

The renin–angiotensin system (RAS) plays a crucial role in regulating blood pressure and the cardio-renal system. The classical RAS, mainly mediated by angiotensin I, angiotensin-converting enzyme, and angiotensin II, has been reported to be altered in critically ill patients, such as those in vasodilatory shock. However, recent research has highlighted the role of some components of the counterregulatory axis of the classical RAS, termed the alternative RAS, such as angiotensin-converting Enzyme 2 (ACE2) and angiotensin-(1–7), or peptidases which can modulate the RAS like dipeptidyl-peptidase 3, in many critical situations. In cases of shock, dipeptidyl-peptidase 3, an enzyme involved in the degradation of angiotensin and opioid peptides, has been associated with acute kidney injury and mortality and preclinical studies have tested its neutralization. Angiotensin-(1–7) has been shown to prevent septic shock development and improve outcomes in experimental models of sepsis. In the context of experimental acute lung injury, ACE2 activity has demonstrated a protective role, and its inactivation has been associated with worsened lung function, leading to the use of active recombinant human ACE2, in preclinical and human studies. Angiotensin-(1–7) has been tested in experimental models of acute lung injury and in a recent randomized controlled trial for patients with COVID-19 related hypoxemia. Overall, the alternative RAS appears to have a role in the pathogenesis of disease in critically ill patients, and modulation of the alternative RAS may improve outcomes. Here, we review the available evidence regarding the methods of analysis of the RAS, pathophysiological disturbances of this system, and discuss how therapeutic manipulation may improve outcomes in the critically ill.

## Introduction

The renin–angiotensin system (RAS) is a complex regulatory system involved in the control of blood pressure and physiology of the cardio-renal system. The classical RAS, main effector of the system, is mediated by angiotensin-converting enzyme (ACE) and angiotensin II (Ang II). Hypoperfusion, sympathetic activation, or hypoxic metabolism trigger the release of renin from the juxtaglomerular apparatus, which cleaves angiotensinogen (produced by the liver) into angiotensin I (Ang I) [1]. ACE, primarily found in the membrane-bound endothelium of the pulmonary and renal capillary beds, converts Ang I into Ang II [2]. Specific effects of Ang II are mediated by the activation of the angiotensin II receptor type 1 (AT_1_), widely distributed in the liver, adrenals, brain, lung, kidney, heart, and vasculature [3], which results to vasoconstriction, pro-inflammatory effects, fibrosis, vasopressin, and aldosterone release [4–8].

In critically ill patients, especially those with vasodilatory shock, the classical RAS may be pathophysiologically affected [9]. Activation of the RAS is observed and characterized by increased levels of renin and Ang I, along with impaired Ang II signaling, resulting in an elevated circulating Ang I / Ang II ratio, which has been linked to mortality [9–11]. Additionally, disturbances in Ang II signaling can occur at the tissue level, such as a reduction in AT_1_ expression in blood vessels and kidneys in sepsis models [12, 13].

While there is growing evidence of classical RAS alterations in critically ill patients, recent research has also highlighted disturbances in the counterbalancing axis of the classical RAS: the alternative RAS. The main functions observed so far in the alternative RAS are its ability to degrade Ang II via the activity of angiotensin-converting enzyme 2 (ACE2). The classical RAS can also be modulated through the action of peptidases, such as dipeptidyl peptidase 3 (DPP3), which can degrade peptides from both the classical and alternative RAS [14, 15].

Here, we provide an overview of the physiology and alterations of the alternative RAS and DPP3 in critically ill patients and discuss potential therapeutic strategies targeting this system.

## Alternative renin–angiotensin system physiology

**Fig. 1:**
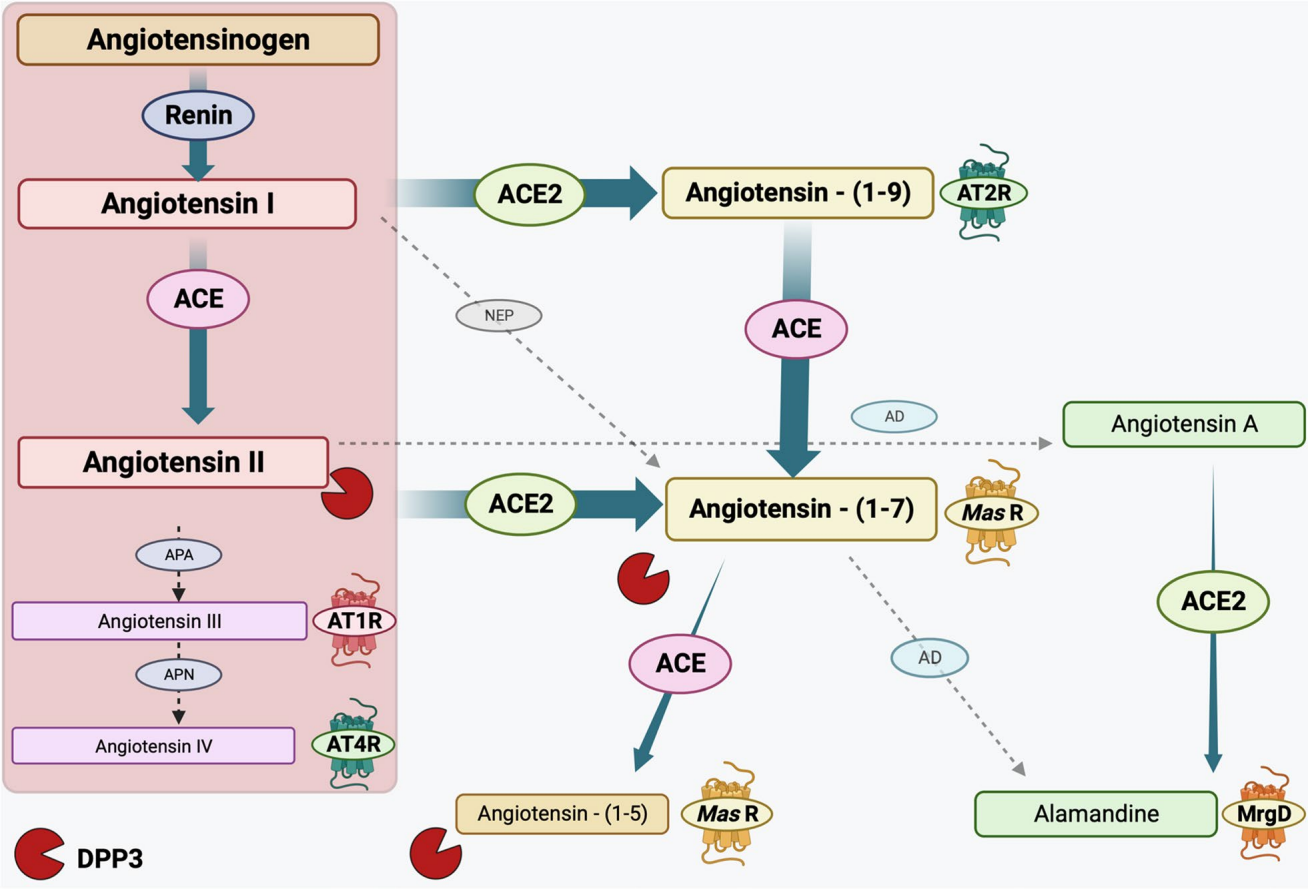
Classical and alternative renin–angiotensin system. alamandine; angiotensin-(1-7), angiotensin-(1-9), angiotensin III; angiotensin IV; MasR: Mas receptor; APA (aminopeptidase A), APN (alanyl aminopeptidase N), angiotensin converting enzyme 33 2 (ACE2), AT2 (angiotensin II receptor 2), AT4 (angiotensin II receptor 4), MrgD (Mas-related G protein-coupled receptor member D), neprilysin (NEP).

The alternative RAS is mostly mediated by angiotensin-(1–7) (abbreviated to Ang-(1–7)). It is associated with various counterbalancing effects on the classical RAS, including anti-inflammatory, anti-fibrotic, and vasodilatory effects [16]. Ang-(1–7) is cleaved from Ang II through the action of ACE2 [17]. ACE2 either directly converts Ang II to Ang-(1–7) or converts Ang I to angiotensin-(1–9) (Ang-(1–9)), which is then further converted to Ang-(1–7) by ACE [18]. Ang-(1–7) can also be produced directly from Ang I through the action of peptidase, such as NEP (neprilysin) [19] (Fig. 1).

Ang-(1–7) can bind to the Mas Receptor (MasR). MasR is a proto-oncogene, G protein-coupled receptor located in the brain, heart, kidney, and other tissues. Its expression is particularly high in the cardiovascular system, where its activation can induce vasodilation through the release of nitric oxide (NO), a decrease in baroreflex sensitivity, sympathetic tone, blood pressure, cardiac hypertrophy, and fibrosis [14, 20–25]. In the brain, MasR can have neuroprotective effects and play a role in cognitive function [14]. MasR has been also described to colocalize with AT_2_, and an interaction between both receptors has also been reported [26]. Ang-(1–7) might induce the heterodimerization of MasR and AT_2_, potentially being responsible for functional effects [27, 28].

On the other hand, Ang-(1–9) can bind AT_2_ and increase baroreflex sensitivity, increase parasympathetic tone, reduce cardiac hypertrophy, fibrosis, and inflammation [29–31]. The activation of AT_2_ also induces vasodilatation, NO release, and natriuresis [29, 32].

Further processing of Ang-(1–7) can form two biologically active compounds: angiotensin-(1–5) (Ang-(1–5)) and alamandine ([Ala1]-Ang-(1–7)). Ang-(1–5) is produced from Ang-(1–7) through the activity of ACE [33], while alamandine ([Ala1]-Ang-(1–7)). is generated from Ang-(1–7) by the action of aspartate decarboxylase (AD) or by the conversion of Ang II to angiotensin A ([Ala1]-Ang II), which is then converted to alamandine ([Ala1]-Ang-(1–7)) by ACE2 [34]. Alamandine ([Ala1]-Ang-(1–7)) binds to the Mas-related G protein-coupled receptor member D (MrgD), leading to similar effects as Ang-(1–7) [35]. Alamandine ([Ala1]-Ang-(1–7)) can induce endothelium-dependent vasorelaxation by activating its receptor [34], and has been associated with cardioprotective effects [36].

Angiotensin III (Ang III) and IV (Ang IV) are also products of Ang II degradation. Ang III is generated by the cleavage of Ang II through the action of the aminopeptidase A [37]. Ang III activates both AT_1_ and AT_2_ receptors, producing similar effects to Ang II [38]. Ang IV is produced from Ang III by the action of another peptidase (alanyl aminopeptidase N) and can interact with the angiotensin II-receptor 4 (AT_4_), which has been identified as the transmembrane enzyme insulin-regulated membrane aminopeptidase (IRAP), and has been shown to induce cardioprotective effects, increase renal cortical blood flow and natriuresis, stimulate endothelial NO synthase, and promote vasodilation; effects that might be related to IRAP inhibition rather than receptor activation [37, 39]. Ang IV has been reported to also induce AT_1_ activation at higher doses compared to Ang II [39]. However, further studies are needed to determine their exact roles in acute or chronic illnesses.

DPP3 is a cytosolic, zinc-dependent metalloprotease found ubiquitously in human cells [40]. It plays a role in regulating the immune response and oxidative stress under normal conditions [41]. In human HK-2 renal epithelial cells, DPP3 can hydrolyze Ang-(1–7) into Ang-(3–7) and rapidly convert Ang-(3–7) into Ang-(5–7) and the inhibition of DPP3 is associated with increased intracellular levels of Ang-(1–7) [42]. Despite its primarily intracellular location, circulating DPP3 has been detected among healthy blood donors [43]. It is hypothesized to be released during a cell death process in conditions as shock, where it is responsible for the cleavage of various peptides, including enkephalins, endorphins, and angiotensin peptides that are less than 10 residues in length such as Ang II, Ang-(1–7), or Ang-(1–5) [15].

In summary, renin cleaves angiotensinogen into Ang I, which is further transformed into Ang II through the action of ACE through the classical RAS. In the alternative RAS, ACE2 metabolizes Ang II into Ang-(1–7). Ang II binds to both AT_1_ and AT_2_ while Ang-(1–7) interacts with MasR and AT_2_, causing effects that counterbalance the ACE/Ang II/AT_1_ axis. DPP3 release can modulate the RAS, while enhanced ACE2 activity shifts the balance towards the ACE2/Ang-(1–7) axis.

## Methods to assess the circulating RAS

Immunological assays, such as radioimmunoassay or enzyme-linked immunosorbent (ELISA), and mass spectrometry are commonly used for angiotensin quantification. However, analyzing the peptides of the RAS can be challenging, as errors can be associated with the sampling and quantification process. First, in the absence of enzyme inhibitor cocktails during sample processing, peptides of the RAS can be further metabolized by RAS enzymes after blood sampling. As an example, blood sampling with the addition of EDTA to collect plasma can inhibit metallopeptidases such as aminopeptidases, ACE, or ACE2, enabling the analysis of Ang II or Ang-(1–7). However, the residual renin activity might increase Ang I levels, potentially leading to misinterpretation [44].To counteract this limitation, the use of a liquid chromatography-mass spectrometry method, known as *equilibrium analysis*, without the need for the use of enzyme inhibitor cocktails has been reported [45]. Another limitation in quantifying angiotensin is the low circulating concentration of most RAS peptides, along with the fact that the majority of the enzymes involved in RAS modulation are membrane-bound, not quantified by these techniques [44]. The specificity of the method employed for quantification can also contribute to differences in angiotensin quantification. This was highlighted in a study comparing an ELISA method with a radioimmunoassay, in which the ELISA method was associated with increased Ang II and Ang-(1–7) levels, suggesting a cross-reactivity with other peptides with ELISA [46]. Similar results were reported in studies assessing angiotensin peptides concentration in severe COVID-19, with increased levels of Ang II and Ang-(1–7) in studies using ELISA compared to liquid chromatography–mass spectrometry [47].

Therefore, careful assessment of the methodology employed for angiotensin quantification, from blood sampling to the quantification method, is crucial for accurate interpretation of RAS analysis reported in the literature.

## The alternative renin–angiotensin system in shock

### Sepsis and vasodilatory shock

A dysfunction of classical RAS signaling is the primary alteration observed in sepsis and vasodilatory shock. Increased renin levels and an elevated Ang I/Ang II ratio have been reported and are correlated with worse outcomes [9–11].

The main hypothesis proposed to explain the imbalance in the Ang I/Ang II ratio is a decrease in ACE activity [9]. This hypothesis is supported by a study involving 72 pediatric patients with septic shock, where circulating ACE activity was measured in serum using an ELISA method. In this study, 69% of the patients presented undetectable ACE activity. Furthermore, ACE activity was associated with a composite outcome of AKI, renal replacement therapy, or 28-day mortality. Interestingly, ACE concentration was increased, suggesting a release of inactive endothelial ACE into the circulation secondary to endothelial injury or ACE inhibition [48]. However, issues with the method used to assess ACE activity in this study could have contributed to the observed results, similarly to the interference of the fluorescent ACE2 assay with human serum or serum albumin in COVID-19 [49]. Finally, downregulation of AT_1_ in blood vessels, heart, and kidneys has been reported in sepsis [12, 13], resulting in a deficiency in Ang II signaling at a tissue level. All these observations have led to increased interest in the use of angiotensin II as a vasopressor [10, 50, 51].

However, there is evidence suggesting that the alternative RAS may be activated during sepsis and vasodilatory shock and could be a therapeutic target in selected patients [52, 53].

#### Preclinical studies

Expression of renal tubular ACE2 and MasR has been reported to be increased in a mouse model of lipopolysaccharide (LPS)-induced AKI, suggesting possible activation of the ACE2/Ang-(1–7)/MasR axis during sepsis [54], a hypothesis that should be interpreted with caution as high enzyme concentrations (ACE) are not necessarily associated with increased enzyme activity [48].

In support to a protective role of ACE2/Ang-(1–7) axis, preclinical studies have tested the administration of Ang-(1–7) in different models. In a LPS model, Ang-(1–7) administration led to improved renal function with decreased levels of urea, creatinine, and cystatin C. Additionally, there was a reduction in the rise of inflammatory cytokines such as tumor necrosis factor (TNF)-α, IL-1, and IL-6 in both serum and kidney [55]. These effects, primarily mediated through MasR stimulation, may also involve AT_2_, as evidence suggests that AT_2_ agonists can decrease tissue levels of neutrophil gelatinase-associated lipocalin (NGAL) and kidney injury molecule 1 (KIM-1) following LPS challenge [56]

In a rat model of sepsis, treatment with 1 mg/kg of Ang-(1–7) at 3 and 6 h after CLP improved arterial pressure, organ function and survival, from 36.4% to 83.3% at 24 h [57]. Ang-(1–7) modulated the immune response, and reduced oxidative stress and apoptosis [57]. In a large animal experimental septic shock model in sheep, early treatment with high doses of Ang-(1–7) improved hemodynamic status. Moreover, Ang-(1–7) achieved normal arterial lactate levels in 6/7 animals at the end of the experiment; reduced norepinephrine requirements; prevented renal dysfunction, and attenuated the rise in interleukin-6. A trend to better survival was also observed, with 2 deaths before 24 h in the control group, while all the 7 treated animals survived [58].

AT_2_ has also been reported to be downregulated in adrenal glands in experimental LPS sepsis [59]. However, few studies reported a beneficial effect of AT_2_ agonist in sepsis [56, 60].

Finally, procizumab, a monoclonal antibody designed to inhibit circulating DPP3 activity in order to limit the degradation of peptides of the RAS, has been tested in a preclinical model of sepsis induced by CLP. Treatment was administered 16 h after CLP and was associated with restored systolic dysfunction from 39 to 51% within 30 min, reduced oxidative stress in the heart, measured by dihydroethidium staining, and improved survival from 63 to 83% (p = 0.0026), offering potential for a new targeted therapy [61].

#### ***Clinical ***studies

In humans, a prospective observational study assessed the relationship between urinary ACE2 activity and AKI in critically ill patients. The study analyzed urinary ACE2 activity in 105 patients at risk of AKI. Of these, 30% had sepsis. The main outcome was severe AKI. The study found that, within 12 h of inclusion, 30% of patients had developed AKI. Patients without AKI had significantly higher uACE2 activity compared to those with AKI at 12 h. The authors proposed a pathophysiological hypothesis of upregulation of the alternative RAS to induce vasodilation, increase renal blood flow, glomerular filtration, natriuresis, and diuresis, while downregulating inflammatory and profibrogenic pathways [62].

Up to now, no study has assessed circulating ACE2 concentration, activity or levels of Ang-(1–7) in humans during sepsis.

DPP3 can be released into the circulation and modulate both the classical and alternative RAS, contributing to its imbalance [15, 63]. In a prospective observational international study involving 585 patients with sepsis, high admission DPP3 levels were associated with an increased incidence of AKI within 7 days, greater use of renal replacement therapy (RRT), longer ICU stay, and higher mortality rates [64].

### Non-vasodilatory shock

High DPP3 levels, which are associated with increased mortality, are not limited to vasodilatory shock but may be observed also in cardiogenic and hemorrhagic shock. An ancillary analysis of the FROG-ICU study, a prospective observational multicenter cohort study conducted in France and Belgium, included 422 (64%) patients admitted for septic shock, 136 (20%) for cardiogenic shock, and 107 (16%) for hemorrhagic shock. DPP3 levels above the median were associated to higher mortality rates at day 28, both in the overall population and across different types of shock. Furthermore, patients who developed AKI and required RRT showed higher levels of DPP3 [65]. Similarly, DPP3 levels at day 1 and 2 were associated with AKI and 28-day mortality in a mixed ICU cohort of 650 adults [66].

### Severely ill burn patients

DPP3 has been associated with illness severity in burn patients. In a prospective two-center cohort study, DPP3 levels at admission were significantly higher in non-survivors compared to survivors at 90 day, and persistent increases in DPP3 levels were associated with worse outcomes. DPP3 levels were linked to cardiovascular failure within the first 48 h, and there was a significant elevation of DPP3 in a small subset of patients with systolic left ventricular dysfunction compared to those without. Additionally, patients with AKI had higher DPP3 levels compared to those without AKI [67].

**Fig. 2:**
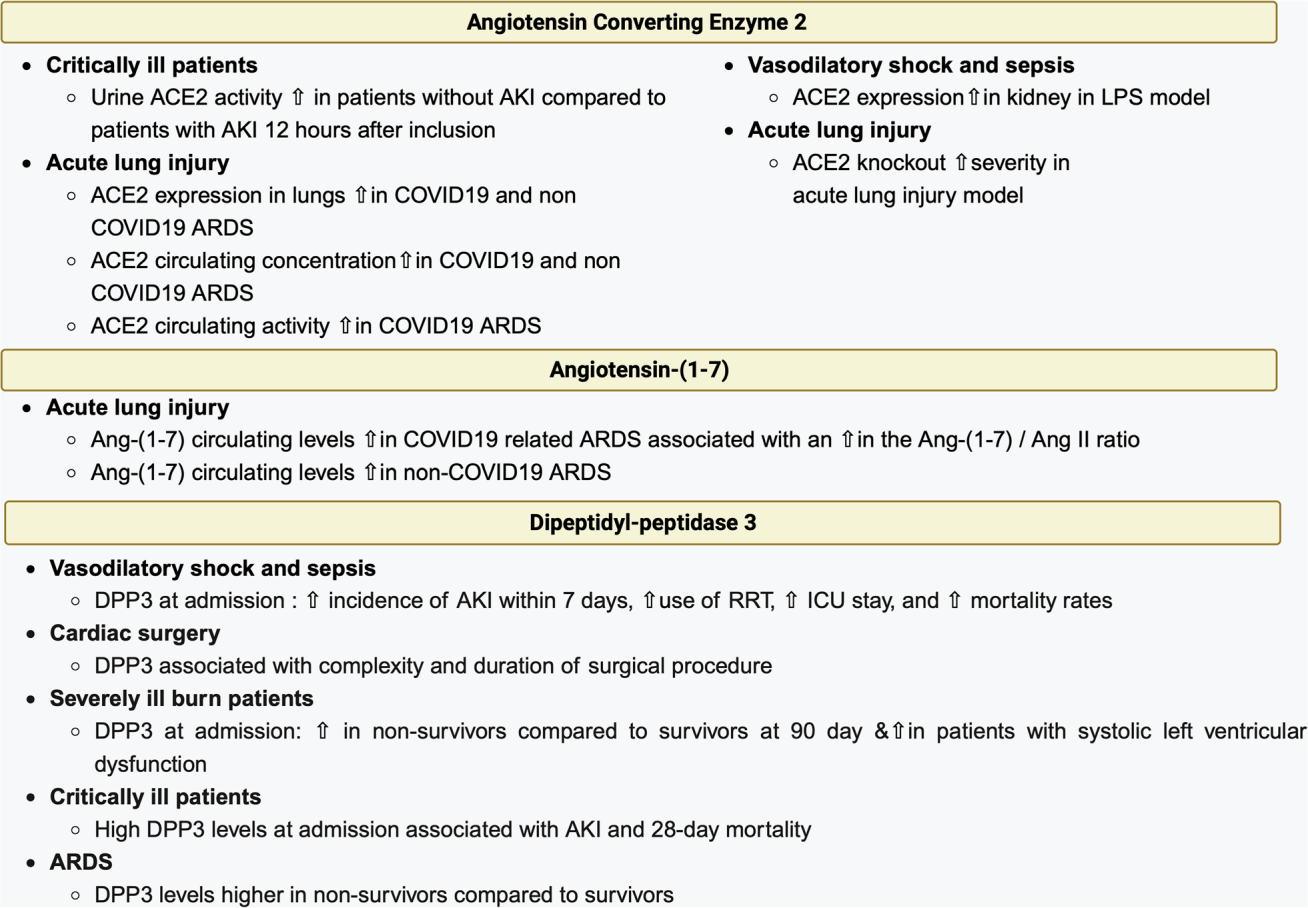
Alterations in the alternative renin–angiotensin system in critically ill patients. ACE2: angiotensin converting enzyme 2; AKI: acute kidney injury; ARDS: acute respiratory distress syndrome; DPP3: dipeptidyl peptidase 3

Altogether, activation of the ACE2/Ang-(1–7) axis during shock could be interpreted as a response to injury, with its activation leading to a reduction in the inflammatory response observed during sepsis (Fig. 2).

This highlights the potential for therapeutic strategies aimed at modulating RAS equilibrium in sepsis, including the administration of exogenous Ang II, rhACE2 or Ang-(1–7), as well as DPP3 inhibition (Table 1).

**Table 1:** Therapies targeting the alternative renin–angiotensin system

Therapies targeting the alternative renin–angiotensin system			
*Preclinical studies*			
	Study	Model	Main findings
rhACE2	Imai et al. 2005	Acid-induced acute lung injury in mice	Recombinant human ACE2 reduced changes in elastance observed in the lung and wet to dry ratio induced by acid injury
Angiotensin-(1–7)	Zhu et al. 2021	LPS in mice	Ang-(1–7) administered for 15 days, followed by an LPS challenge, resulted in reduced increase in TNF-α, IL-1, and IL-6 in both serum and kidney compared to the LPS group, and to reduced levels of urea, creatinine, and cystatin C
	Tsai et al. 2018	CLP in rat	Administration of Ang-(1–7) at a dosage of 1 mg/kg at 3 and 6 h after CLP led to a reduced increase in IL-6, improved mean arterial pressure, and enhanced organ function, as evidenced by liver and renal biomarkers. Additionally, survival rates improved from 36.4% to 83.3% at 24 h (p < 0.01)
	Garcia et al. 2023	Polymicrobial peritonitis in sheep	Ang-(1–7) started at sepsis induction prevented the development of septic shock, with 6 out of 7 animals maintaining normal arterial lactate levels at 24 h. Ang-(1–7) reduced norepinephrine requirements and prevented renal dysfunction. Additionally, there was a reduction in the increase of IL-6, associated with a trend toward improved survival rates. In the control group, 2 out of 7 animals died before 24 h, while all 7 animals in the treated group survived
	Zambelli et al. 2015	Acid-induced acute lung injury in rats	High doses of Ang-(1–7) improved oxygenation, reduced white blood cells counts and reduced inflammatory cell numbers in bronchoalveolar lavage
AT_2_ agonist	Fatima et al. 2021	LPS in mice	A single dose of C21, an agonist of AT_2_, resulted in an increase in the circulating anti-inflammatory cytokine IL-10 and a reduction in the biomarkers of AKI, NGAL and KIM1
	Shih et al. 2023	CLP in rats	CGP42112 three hours after the CLP procedure mitigated hypotension, led to a reduction in the increase of biomarkers for liver and renal injury, and improved survival rates from 20 to 50% at 24 h (p < 0.05)
DPP3 inhibition	Deniau et al. 2020	CLP in rats	Procizumab, a humanized antibody that neutralizes DPP3 activity, administered 16 h after CLP, restored systolic dysfunction from 39 to 51% within 30 min. It also reduced oxidative stress in the heart and improved survival rates from 63 to 83% (p = 0.0026) at 120 min after randomization
*Clinical studies*			
	Study	Patients	Main findings
rhACE2	Zoufaly et al. 2020	45 years old woman with severe COVID19	rhACE2 was started 9 days after symptoms onset twice daily and resulted in reduction in circulating Ang II levels and increase in Ang-(1–7), Ang-(1–9) and Ang-(1–5)
	NCT04335136	181 hospitalized patients for COVID19	The primary endpoint was a composite endpoint of all cause-death or invasive mechanical ventilation up to 28 days or hospital discharge. No differences were reported in the primary outcome
Angiotensin-(1–7)	Martins et al. 2021	28 patients admitted to the ICU	Ang-(1–7) was administrated intravenously for up to 7 days. No changes in hemodynamic parameters were observed. Bradycardia was observed in one patient
	Self et al. 2023	343 patients with hypoxemia	Patients were randomized to receive daily either 0.5 mg/kg of angiotensin-(1–7) for 5 days or placebo. Oxygen-free days did not differ between the groups at day 28 (mean difference − 2.3 days [95% CrI, − 4.8 to 0.2]

## The alternative renin–angiotensin in cardiac surgery

### Cardiac surgery

There have been only limited studies investigating the RAS in the context of cardiac surgery. In a prospective monocentric study involving 197 patients who underwent cardiac surgery, individuals with a greater increase in serum renin concentrations from baseline after surgery had a higher likelihood of developing vasoplegia and AKI [68]. The investigators hypothesized that reduced angiotensin II levels or a lack of signaling through the AT_1_ could lead to an increase in renin levels, similar to that is observed in vasodilatory shock [69].

DPP3 levels were reported in a monocentric study involving 203 patients who underwent cardiac surgery, categorized as minimally invasive (*n* = 22), elective (*n* = 166), or emergency (*n* = 15). The study found that DPP3 levels were elevated on day 1 and strongly correlated with the complexity and duration of the surgical procedure, suggesting a potential association with direct tissue injury. Furthermore, higher DPP3 levels on day 2 were linked to prolonged use of vasopressors, an increased risk of AKI, and an extended stay in the intensive care unit [70].

However, no studies have examined the peptides of the alternative RAS in the context of cardiac surgery.

### The alternative renin–angiotensin system in acute lung injury

#### Preclinical studies

Preclinical studies have highlighted a protective role of the ACE2/Ang-(1–7)/MasR axis. In an experimental study where mice lacking the ACE2 gene underwent a CLP procedure, ACE2 knockout mice experienced increased mortality, significant increase in lung elastance, pulmonary edema, and leukocyte accumulation compared to wild-type mice [71]. The protective role of ACE2 was further supported by the use of active recombinant human ACE2 (rhACE2) protein as a rescue therapy for acid-induced acute lung injury (ALI). rhACE2 was associated with a decrease in the severity of experimental ALI, as evidenced by reduced changes in lung elastance and a decreased wet-to-dry ratio compared to placebo-treated mice [71].

The potential of the ACE2/Ang-(1–7) axis as a treatment for ALI was also assessed in an experimental study involving rats with hydrochloric acid-induced ALI. This study found that Ang-(1–7) administration reduced the decline in arterial oxygenation, and decreased leukocyte and polymorphonuclear counts in broncho-alveolar lavage. However, no significant difference was observed in alveolar inflammatory cytokines. Additionally, treatment with Ang-(1–7) was associated with a decrease in circulating white blood cell count and a reduction in collagen deposition in the lungs [72].

#### ***Clinical ***studies

Based on these data suggesting a protective role of the alternative RAS, human studies have analyzed the ACE2/Ang-(1–7) axis in patients with acute respiratory distress syndrome (ARDS). An activation of the alternative RAS was reported, characterized by increased ACE2 expression in the lungs, elevated circulating ACE2 concentrations and activity, along with an increase in Ang-(1–7) circulating levels.

ACE2 expression was observed to be upregulated in endothelial cells in the lungs, together with an increase in circulating ACE2 concentration in both COVID19- and non-COVID19-related ARDS [73]. Moreover, a natural substrate conversion assay of Ang II to Ang-(1–7), quantified with a liquid-chromatography mass spectrometry method, demonstrated that circulating ACE2 activity was elevated in COVID-19-related ARDS. This increase in ACE2 activity was associated with a rise in circulating Ang-(1–7) levels and Ang-(1–7) / Ang II ratio, indicating a shift towards the alternative RAS [45]. Similarly, using the same mass spectrometry method, equilibrium Ang-(1–7) levels were shown to be increased in non-COVID19-related ARDS as well [74].

The use of the ACE2/Ang-(1–7) axis as a therapeutic strategy has been also tested in clinical studies. rhACE2 was tested in an ARDS pilot trial and in a case report for severe COVID-19-related ARDS. In both studies, rhACE2 was associated with changes in the RAS, with a decrease in Ang II concentration and increased Ang-(1–7) and Ang-(1–5) levels [75, 76]. Additionally, rhACE2 was investigated in a randomized controlled trial involving hospitalized patients with COVID-19. However, the primary endpoint (all-cause death or invasive mechanical ventilation up to 28 days or hospital discharge) was not significantly different between groups (NCT04335136).

Ang-(1–7) has been also tested in this situation. A pilot study was conducted to assess the safety of a pharmaceutically formulated Ang-(1–7) (TXA-127) for the treatment of severe COVID-19. COVID-19 patients requiring oxygen were randomized to receive either TXA-127 or a placebo and no side effects were reported [77]. In another phase 1 clinical trial involving 28 intensive care unit (ICU) patients with severe COVID-19-related ARDS, no vasodilatory effect was found, and no significant changes in circulating RAS levels were observed during the study [78]. Two multicenter randomized controlled trials recently explored the use of angiotensin-(1–7) (TXA-127) and angiotensin II receptor 1-biased ligand (TRV-027) in severe COVID-19 [79]. In the TXA-127 study, 343 patients with hypoxemia were randomized to receive daily either 0.5 mg/kg of angiotensin-(1–7) for 5 days or placebo. The primary outcome—oxygen-free days—did not differ between the groups at day 28 (mean difference − 2.3 days [95% CrI, − 4.8 to 0.2]; adjusted OR, 0.88 [95% CrI, 0.59 to 1.30]). However, limitations of this study were suggested to include a high administered dose, the lack of quantification of the RAS components, and the method of administration of the peptide as a 3-h bolus instead of a continuous infusion [80]. The TRV-027 trial randomized 290 patients to receive either an AT_1_ biased ligand or placebo. The average number of oxygen-free days did not differ significantly between the groups, with 8.1 days in the treatment group compared to 10.5 days in the control, adjusted OR 0.74 (0.48 to 1.13) [79].

Given that DPP3 can cleave RAS peptides and that Ang II levels were reported to be reduced in COVID-19 patients with ARDS compared to healthy controls [81], DPP3 was examined in a European multicenter prospective cohort study of 80 critically ill COVID-19 patients. The primary hypothesis was that DPP3 may be released by injured pulmonary cells and may contribute to the RAS disturbance. Among these, 82.5% required invasive mechanical ventilation, and had a median PaO_2_/FiO_2_ ratio of 146. Acute kidney injury (AKI) occurred in 42.5% of patients, and 47.5% received vasopressors. DPP3 levels at admission were higher in non-survivors compared to survivors at day 28, a difference becoming more pronounced at day 3 and day 7 [82]. As DPP3 can cleave both the classical and alternative RAS, its inhibition may improve endogenous Ang II levels, with potential beneficial effects on pulmonary function [83] and/or increase Ang-(1–7) levels [63].

Altogether, these data support that ACE2 and Ang-(1–7) contribute to the lung injury response and that a shift towards the alternative RAS occurs in response to injury. The activation of the ACE2/Ang-(1–7) axis might represent a protective adaptative mechanism. In this context, targeting the alternative RAS, through activation of the ACE2/Ang-(1–7)/MasR axis or DPP3 inhibition, may represent a future therapeutic strategy. However, further studies are needed, as this approach failed to improve outcomes in COVID-19 patients with hypoxemia in a recent randomized controlled trial [79].

### Relation between alternative renin–angiotensin system and classical renin–angiotensin system

The REMAP-CAP study tested the hypothesis that pharmacological inhibition of the classical ACE/Ang II RAS with an ACE inhibitor or an angiotensin receptor blocker (ARB) could improve the outcomes for severe COVID-19 patients [84]. The study randomized 679 critically ill patients to receive either ACE inhibitor, ARB, or placebo and was stopped due to safety concerns, as patients receiving ACE inhibitors or ARBs showed a higher probability of reduced organ support-free days. Interpretation of this study concerning its relation to the alternative RAS remains hypothetical since no analysis of the RAS peptides has been reported in this setting.

However, severe COVID-19 has been reported in other studies to be associated with a decrease in ACE activity and reduced Ang II/Ang I ratio, suggesting a defect in the classical RAS, associated with an increase in ACE2 activity [74]. In this situation, pharmacological inhibition of the ACE/Ang II axis could have worsened the defect in the classical RAS observed during COVID-19. Similarly to what is observed during vasodilatory shock, the restoration of Ang II signaling could represent another strategy in this condition, that could, through increased ACE2 activity, be associated with increased Ang-(1–7) levels. However, the different evolution of the peptides of the RAS under therapeutic modulation remains hypothetical and will need further analysis [80].

## Conclusions

Although extensive research has focused on the classical RAS in critically ill patients, recent advances have also highlighted the importance and complexity of the alternative RAS.

Predominantly mediated by ACE2 and Ang-(1–7), the alternative RAS appears to play a protective role in various critical conditions. Decreased ACE2 activity has been associated with acute lung injury, while treatment with recombinant human ACE2 has shown promising protective effects in experimental models. Nonetheless, the comparative efficacy of rACE2 treatment versus an ACE inhibitor remains uncertain, given that both therapies aim to reduce angiotensin II and increase Ang-(1–7). Administration of Ang-(1–7) appears protective in models of acute lung injury, or sepsis. However, it failed to improve oxygen-free days in a recent randomized controlled trial. Additionally, DPP3, an enzyme involved in RAS degradation, has been implicated in various pathological states and could be a future target of therapy.

The alternative RAS presents a promising avenue for therapeutic strategies in critically ill patients. Further studies are needed to enhance our understanding of its physiology and pathophysiology and to explore potential therapeutic interventions based on the alternative RAS. Such interventions could have significant implications for the management and outcomes of critically ill patients.

## Data Availability

Not applicable.

## Abbreviations

ACE: Angiotensin-converting enzyme
ACE2: Angiotensin-converting enzyme 2
AD: Aspartate decarboxylase
AKI: Acute kidney Injury
ALI: Acute lung Injury
Ang-I: Angiotensin I
Ang-II: Angiotensin II
Ang-III: Angiotensin III
Ang-IV: Angiotensin IV
Ang-(1–5): Angiotensin 1–5
Ang-(1–7): Angiotensin 1–7
Ang-(1–9): Angiotensin 1–9
ARDS: Acute respiratory distress syndrome
AT_1_: Angiotensin II receptor 1
AT_2_: Angiotensin II receptor 2
AT_4_: Angiotensin II receptor 4
COVID-19: Coronavirus disease 2019
DPP3: Dipeptidyl-peptidase 3
ICU: Intensive care unit
KIM-1: Kidney injury molecule 1
LPS: Lipopolysaccharide
MrgD: Mas-related G protein-coupled receptor member D
NGAL: Neutrophil gelatinase-associated lipocalin
RAS: Renin–angiotensin system
rhACE2: Human recombinant ACE2
RRT: Renal replacement therapy
